# Effect of copper oxide nanoparticles and light-emitting diode irradiation on the cell viability and osteogenic/odontogenic differentiation of human stem cells from the apical papilla

**DOI:** 10.1186/s12903-023-02916-0

**Published:** 2023-04-28

**Authors:** Hamed Karkehabadi, Afsaneh Rahmati, Roshanak Abbasi, Abbas Farmany, Rezvan Najafi, Rooholah Behroozi, Loghman Rezaei-soufi, Hadiseh Abbaspourrokni

**Affiliations:** 1 Department of Endodontics, Dental Research Center, Dental School, Hamadan University of Medical Sciences, Hamadan, Iran; 2 Department of Endodontics, Dental School, Hamadan University of Medical Sciences, Hamadan, Iran; 3 Dental Research Center, Dental School, Hamadan University of Medical Sciences, Hamadan, Iran; 4 Department of Medical Molecular & Genetics, Faculty of Medicine, Hamadan University of Medical Sciences, Hamadan, Iran; 5 Private Dental Practice, Endodontist, Hamadan, Iran; 6 Department of Operative Dentistry, Dental Research Center, Dental School, Hamadan University of Medical Sciences, Hamadan, Iran; 7 Department of Endodontics, Faculty of Dentistry, Mazandaran University of Medical Sciences, Sari, Iran

**Keywords:** Mesenchymal stem cells, Copper oxide nanoparticles, Viability, Differentiation, LED

## Abstract

**Objectives:**

This experimental study aimed to assess the effect of copper oxide nanoparticles (CuONPs) and light-emitting diode (LED) irradiation on the cell viability and osteogenic/odontogenic differentiation of human SCAPs.

**Methods:**

After the culture of SCAPs, the effects of different concentrations of CuONPs on cell viability were evaluated by the methyl thiazolyl tetrazolium (MTT) assay after 24 and 48 h, and the optimal concentration was determined (*n* = 12). SCAPs were then divided into four groups based on the type of treatment: (I) no-treatment control group, (II) exposure to CuONPs, (III) LED irradiation (635 nm, 200 mW/cm^2^) for 30 s, and (IV) exposure to CuONPs combined with LED irradiation. CuONPs were synthesized by a green technique, which was based on reduction and simultaneous stability of copper ions by using the pomegranate peel extract. After treatments, the expression of osteogenic/odontogenic markers including dentin sialophosphoprotein (DSPP), bone sialoprotein (BSP), alkaline phosphatase (ALP), and dentin matrix acidic phosphoprotein 1 (DMP1) was evaluated in all four groups using quantitative real-time polymerase chain reaction (PCR) (*n* = 16). Also, osteogenic differentiation of SCAPs was evaluated qualitatively by alizarin red staining (ARS) to assess the matrix mineralization (*n* = 4). SPSS version 18 was used for data evaluation. The Kruskal–Wallis and Mann–Whitney tests were used to compare the groups.

**Results:**

Exposure to 1 µg/mL CuONPs resulted in maximum viability of SCAPs. Concentrations of CuONPs over 10 µg/mL significantly decreased the viability of SCAPs. Real-time PCR showed that the expression of DMP1, BSP, ALP, and DSPP in CuONPs + LED and LED groups was significantly higher than that in CuONPs and control groups at both 24 and 48 h (*P* < 0.05). The density of ARS increased in all experimental groups after 24 h, and in CuONPs + LED and CuONPs groups after 48 h, compared to the control group.

**Conclusion:**

Addition of CuONPs and LED irradiation of SCAPs in the culture medium significantly enhanced their osteogenic/odontogenic differentiation.

## Background

Pulpitis may occur in immature permanent teeth due to the invasion of cariogenic bacteria or trauma, which may gradually proceed and lead to pulp necrosis. In such cases, odontoblasts die and root development is discontinued [1].

Apexification is the conventional treatment for permanent immature teeth with necrotic pulp, which involves the use of calcium hydroxide paste to induce the formation of an apical barrier, or the application of mineral trioxide aggregate as an apical plug [2]. Although apexification treatment has a high success rate, it can lead to abnormal root morphologies, such as formation of calcified tissue in the root canal system. This treatment does not allow root development in longitudinal and transverse dimensions. Thus, eventually, the tooth would have short roots with thin root canal walls, susceptible to fracture at the end of treatment [3].

Since tissue regeneration does not occur in apexification, endodontic regenerative treatment was introduced as a novel modality to treat immature teeth with a necrotic pulp. Endodontic regenerative procedures are based on the concept of regeneration of the dentin-pulp complex, which has the regeneration potential to restore the lost physiological function of the pulp [4]. Dr. Nygaard Ostby in 1961 was the first to discuss the concept of tissue regeneration for this purpose [5]. He showed that blood vessels can be used to induce new tissue formation in an empty root canal space by induction of blood clot formation at the site. Fibrin is responsible for the formation and stabilization of blood clots, which serve as a scaffold for stem cells of the apical papilla (SCAPs) [3]. These stem cells can differentiate into odontoblasts in the presence of the required growth factors [6]. Tissue engineering requires three major components of a scaffold, morphogen growth factor (s), and mesenchymal stem cells for the regeneration of functional tissue or organ [4, 7]. Apical papilla is a tissue that is located near the apex of the roots of immature teeth, acting as a source of stem cells that differentiate into the dentin-synthesizing odontoblasts, which play a significant role in root formation and regenerative endodontic treatments [8]. In dentistry, numerous potential sources of mesenchymal stem cells are available for tissue engineering. SCAPs provide a source of primary odontoblasts that enable the continuation of root development. Due to proximity to periodontal vasculature, they can regenerate a necrotic pulp even in the presence of periradicular infection [3]. These cells are a group of mesenchymal stem cells present in the apical papilla of permanent immature teeth. The proliferation rate of SCAPs is two to three times higher than the rate in dental pulp stem cells in the culture medium [9]. It has been demonstrated that SCAPs have high viability and potency of multipotent differentiation, including odontogenic, osteogenic, neurogenic, and adipogenic differentiation [10].

Biomaterials have an undeniable role in regenerative endodontics. They can serve as part of a temporary scaffold for cell proliferation and new tissue formation in the regeneration process. Biomaterials used for regenerative treatments have different components, including ions and metal compounds. Considering their continuous exposure to cells, they should have certain properties, such as optimal biocompatibility. Biocompatibility is the basis of regenerative treatments and ensures the regenerative, proliferation, and differentiation properties of stem cells. Moreover, they should possess certain mechanical and chemical properties, such as optimal strength and stability, to preserve the primary structure of stem cells [11].

Nanotechnology refers to the design, synthesis, and application of nano-scale (1–100 nm) materials. The unique physical and chemical properties of nanoparticles, especially their small size and top surface/volume ratio, enhance the availability of biological systems and molecules [12]. However, cytotoxicity can limit the application of nanoparticles. Cytotoxicity is highly influenced by the concentration of nanoparticles and the duration of exposure of cells to nanoparticles [13]. It has been shown that zinc ions have increased the expression of osteogenesis markers, such as OCN, ALP, Col1, BMP2, and Runx2 upon initial contact with HMSCs [14]. Copper oxide (CuO) is the simplest member of the family of copper compounds, which have physical properties, such as high thermal superconductivity, electron correlation effects, and spin dynamics [15, 16]. It is thought that this element is capable of inducing the osteogenic differentiation of mesenchymal cells [17]. Also, due to the narrow band gap, it has optimal photovoltaic and photoconductive properties [18]. CuO nanoparticles (CuONPs) improve the viscosity of liquids, increase thermal conductivity, and enhance energy conversion [19]. Also, high concentrations of copper ions may lead to the formation of reactive species and subsequent changes in protein synthesis and DNA transcription in microorganisms. It has been demonstrated that the replacement of some essential ions can inhibit the activity of enzymes and proteins in the structure of bacterial cells with copper ions. This action also leads to the generation of free radicals, and subsequent inhibition of bacterial proliferation [20].

Low-level laser therapy (LLLT) has photo-stimulatory and photo-biomodulatory effects. LLLT also induces cell proliferation, increases cell metabolism and cellular regeneration, and has anti-inflammatory effects. Previously, photobiomodulation has been demonstrated to increase mitochondrial membrane potential (MMP) and adenosine triphosphate (ATP) levels and to decrease oxidative stress in cells. Moreover, LLLT can accelerate dentin formation following exposure to the dentin-pulp complex. Also, it has been speculated that LLLT can increase the proliferation and differentiation of stem cells [21]. The use of metal nanoparticles and photobiomodulation were able to shorten the inflammatory phase, promote angiogenesis and collagen production [22]. It has been demonstrated that harvesting mesenchymal stem cells from gold nanoparticles functionalized substrates induced by infrared photobiomodulation improved stem cell viability and their capacity to differentiate into osteoblasts and adipocytes [23].

To the best of our knowledge, no previous study has evaluated the impact of CuONPs and light-emitting diode (LED) irradiation on SCAPs.This study examined the effects of CuONPs and LED irradiation on the viability and osteogenic/odontogenic differentiation of human SCAPs.

## Materials and methods

### Isolation and culture of SCAPs

Based on the Guidelines for Stem Cell Research and Clinical Translation of the International Society for Stem Cells Research (ISSCR), the experimental study was conducted. The Ethics Committee of Hamadan University of Medical Sciences approved the protocol of this study (IR.UMSHA.REC.1396.745). SCAPs were isolated from the left upper and lower fully impacted third permanent molars of one healthy donor 18 years of age, with an indication for extraction due to orthodontic reasons. It was noted that over two-thirds of the left upper and lower third permanent molar roots had developed. We obtained a written informed consent from the patient prior to the use of the apical papilla tissue. A dental tweezer was used to separate the apical papilla from the apical portion of incompletely developed teeth. Following enzyme digestion, the SCAPs were isolated and cultured in accordance with previously published protocols [24, 25]. Briefly, a solution of 3 mg/mL type I collagenase (Worthlington Biomedical, Lakewood, NJ, USA) and 1 mol/L phosphate-buffered saline (PBS) (Worthlington Biomedical, Lakewood, NJ) to collect the stem cells, after that, transferred to Dulbecco’s modified Eagle’s medium (Gibco, GrandIsland, NY, USA) at 37 °C for 1 h Cells were incubated at 37 °C, 5% CO2, 85% humidity, and the sterile cell culture flasks (SPL Life Science, Gyeonggi-do, South Korea) was supplemented with 15% fresh bovine serum and 1% penicillin and streptomycin. On the basis of the design and sample size of prior in vitro studies [26, 27], In both experimental and control groups, three repetitions were conducted.

### Assuring the stemness of cells

As soon as 80% confluence was reached, the medium was removed from the flask and the cells were twice rinsed with PBS. The medium culture was added to the flask following the detachment of the cells using trypsin/EDTA. A 15-mL Falcon tube was filled with culture medium and cells and centrifuged at 1200 g for 6 min. Following two rinses with PBS, cell sediment was subjected to flow cytometry for the detection of specific stem cell surface markers (CD105 and CD90), as well as hematopoietic cell surface markers (CD45 and CD34). A positive result for mesenchymal cell surface markers was observed in the cells, which did not exhibit the hematopoietic cell surface markers.

### Study group

SCAPs were then divided into four groups based on the type of treatment: (I) no-treatment control group, (II) exposure to CuONPs, (III) LED irradiation (635 nm, 200 mW/cm^2^) for 30 s, and (IV) exposure to CuONPs combined with LED irradiation.

### Preparation of CuONPs

A green method was adopted to synthesize CuONPs by using the pomegranate peel extract in accordance with the previous protocol [28]. X-ray diffraction (XRD), Fourier-transform infrared spectroscopy (FT-IR), and scanning electron microscopy (SEM) were then performed for the characterization of CuONPs.

In order to synthesize copper oxide nanoparticles, pomegranate peels were systematically washed with deionized water and shadow dried for 14 days. After washing 100 mg of pomegranate peels with double distilled water, the peels were sliced and dried with hot air in an oven. When the complete drying process had been completed, these materials were pulverized until fine dust and dissolved in distilled water (10 g dust/100 mL distilled water) in an Erlenmeyer flask before boiling for 10 min at 60 °C. Whatman filter paper No.1 was used for filtration. In order to store the filtrate, it was freeze-dried and kept at a temperature of 4 degrees Celsius.

In a magnetic stirrer at 60° C, 90 mL of an analytical grade solution of cupric sulphate (5 mM) was mixed with 20 mL of filtrate obtained in a magnetic stirrer with deionized water. During storage, the mixture was maintained at room temperature. There was a gradual formation of a brownish-black precipitate at the bottom of the conical flask. Following that, it was dried and stored in order to be used as a green synthesized CuONPs.

To determine the surface chemistry of NPs, fourier transform infrared spectroscopy (FT-IR) was performed with a Perkin 118. According to Chattopadhyay et al. [29], an analysis of the hydrodynamic sizes and zeta potential distribution of nanoparticles was conducted using the Zetasizer-Nano ZS (Malvern, Malvern Hills, U.K.).

X-Ray Diffraction (XRD) Study of green synthesized CuONPs:

The X-ray powder diffraction study of NPs was conducted in solid form. The diffractometer XPERT-PRO was used to determine diffraction patterns (PANalytical Ltd., The Netherlands) according to the method of Das et al., [30].

Copper oxide nanoparticles were analyzed using high resolution scanning electron microscopy to determine their surface morphology and particle size (Hitachi S-3400N).

### Methyl thiazolyl tetrazolium (MTT) assay

In order to assess the effect of CuONPs on cell viability, and selection of the most appropriate concentration, 110^4^ cells were cultured in each well of a 96-well plate (*n* = 12). The plates were incubated in the incubator (Binder, NY, USA) at 37 °C and 96% humidity for 24 h. The cells were then randomly divided into six groups, and 100 µL of the culture medium containing CuO with 1, 10, 100, 200 and 500 µg/mL concentration was added to each well under sterile conditions.

The plates were incubated for 24 and 48 h. After removing the plates from the incubator, 10 mL of the MTT solution and 90 mL of alpha-MEM culture medium containing 10% fetal bovine serum were added to each well, and the plates were incubated again at 37 °C, 95% humidity and 5% CO_2_ for 4 h. The overlaying medium was then gently removed, and 100 mL of dimethyl sulfoxide (Gibco BRL, Grand Island, NY, USA) was added to each well. After dissolution of formazan crystals, the optical density values were read by an ELISA Reader (BioTek, USA) in the wavelength range of 540–690 nm [31].

### LED irradiation

The optimal concentration of CuONPs was first determined by the methyl thiazolyl tetrazolium (MTT) assay. Next, this concentration of CuONPs was added to the cultured cells 30 min prior to LED irradiation. Next, the cells were irradiated with a LED irradiation in a semi-dark room with one empty row between the wells. The Fotosan 630 LED (Fotosan 630, Korea, MDD, CMS Dental Denmark) with an end tip (1 mm^2^ diameter) which produced light at a wavelength of 620–640 nm (85%) with a peak at 630 nm, the intensity of 200 mW/cm^2^ and energy density (fluence) of 4 mJ/cm^2^ was irradiated for 30 s. According to previous studies [32], here is the formula (energy density = power density x irradiation time) and the expected energy level. The first irradiation was performed 24 h after primary cell culture, and the second was performed 48 h later. During all irradiations, the same operator was responsible.

### Assessment of osteogenic/odontogenic differentiation by real-time polymerase chain reaction (PCR)

Osteogenic/odontogenic differentiation was evaluated in each group at 24 and 48 h, with/without LED irradiation. Six wells (6 repetitions) were assigned to each group for assessment at each time point [33].

RNA extraction from the stem cells in the medium was performed using Trizol reagent (Invitrogen, CA, USA). Next, cDNA was synthesized using Superscript II first-strand cDNA synthesis kit (Invitrogen, CA, USA) as instructed by the manufacturer. Reverse transcription PCR was then performed (7500 Fast Real-Time PCR System; Applied Biosystems, Carlsbad, CA, USA) according to the instructions for dentin sialophosphoprotein (DSPP), bone sialoprotein (BSP), alkaline phosphatase (ALP), and dentin matrix acidic phosphoprotein 1 (DMP1).

### Alizarin red staining (ARS)

To assess the odontogenic/osteogenic differentiation of cells, qualitatively, they were seeded in 96-well plates after exposure to CuONPs and LED irradiation (*n* = 4). Next, they were cultured in an osteogenic/odontogenic medium containing 10 mM beta glycerophosphate (Sigma Aldrich, St. Louis, MO, USA), 10 nm dexamethasone (Sigma Aldrich, St. Louis, MO, USA), and 50 mg/mL L-ascorbic acid. The cells were fixed with 4% paraformaldehyde. Next, after 21 days they were rinsed with phosphate buffered saline (PBS) and incubated with 1% alizarin red stain (Sigma Aldrich, St. Louis, MO, USA) at 37 °C for 30 min to identify the formed mineral nodules. Assessments were made using a spectrophotometer (SpectraMax M2) at 562 nm wavelength [34].

### Statistical analysis

Data were analyzed by SPSS version 18 (SPSS Inc., Chicago, IL). The data were analyzed by the Kruskal–Wallis test and Mann–Whitney U post hoc test. P < 0.05 was considered statistically significant.

## Results

### Characterization of CuONPs

**Fig. 1:**
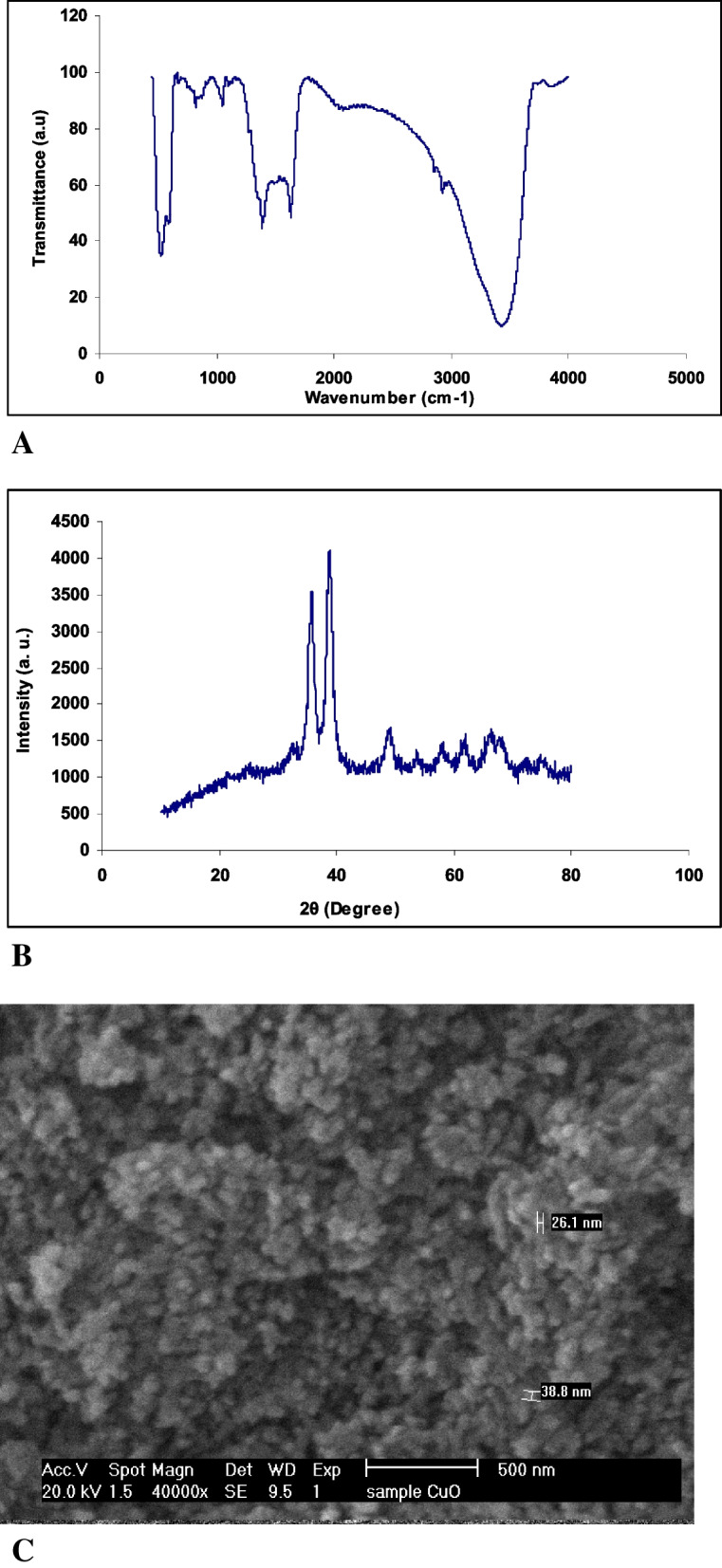
Characterization of nanoparticles. **A**: XRD pattern of CuO nanoparticles, **B**: FT-IR spectra of CuO nanoparticles, **C**: SEM of CuO nanoparticles. **A**: the XRD pattern of CuONPs synthesized from copper nitrate (II) trihydrate and pomegranate peel extract. **B**: the FTIR spectra for the synthesized CuONPs. According to the FTIR spectra of CuONPs, the most significant absorbance peak was noted at 524 cm-1, corresponding to Cu–O stretching vibration. **C**: The surface morphology of the synthesized CuONPs was inspected under the scanning electron microscopy (SEM)

Figure 1A indicates the XRD pattern of CuONPs synthesized from copper nitrate (II) trihydrate and pomegranate peel extract. The synthesis of CuONPs was confirmed by XRD measurements. The XRD peaks at 31.22, 35.87, 38.92, 49.42, 53.87, 58.42, 62.02, 66.82, 68.37, and 73.07 degrees as shown in Fig. 1A belonged to (100), (002), (200), (202), (020), (202), (113), (022), (020), (311), and (004), respectively [30–32]. The observed peaks corresponded to JCPDS card № 048–1548, 048–1548, and 048–1548, which represents CuO mass [35–37].

**Fig. 2:**
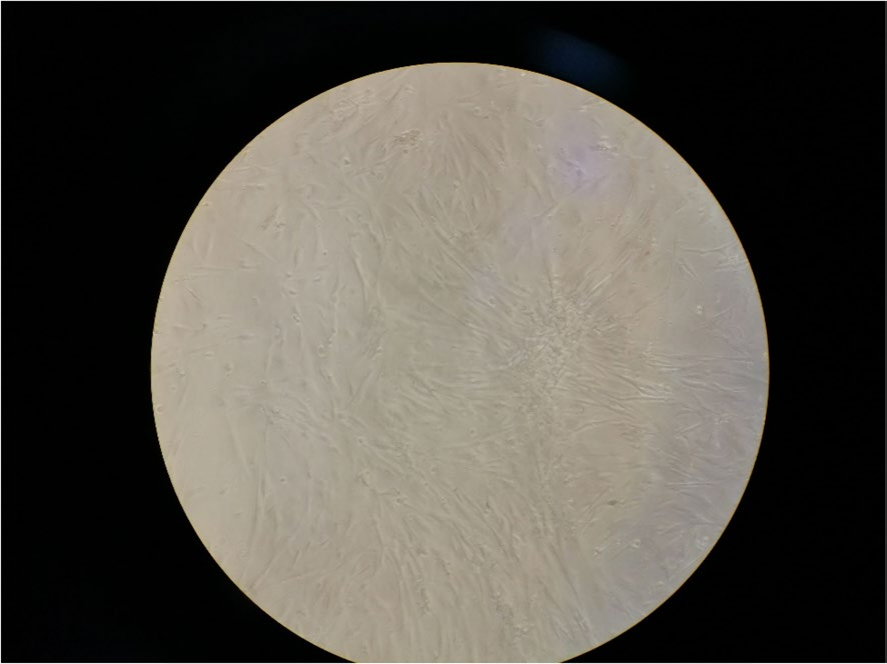
The isolated cells had a fibroblast-like morphology (spindle-shaped) with optimal homogeneity (scale × 10)

**Fig. 3:**
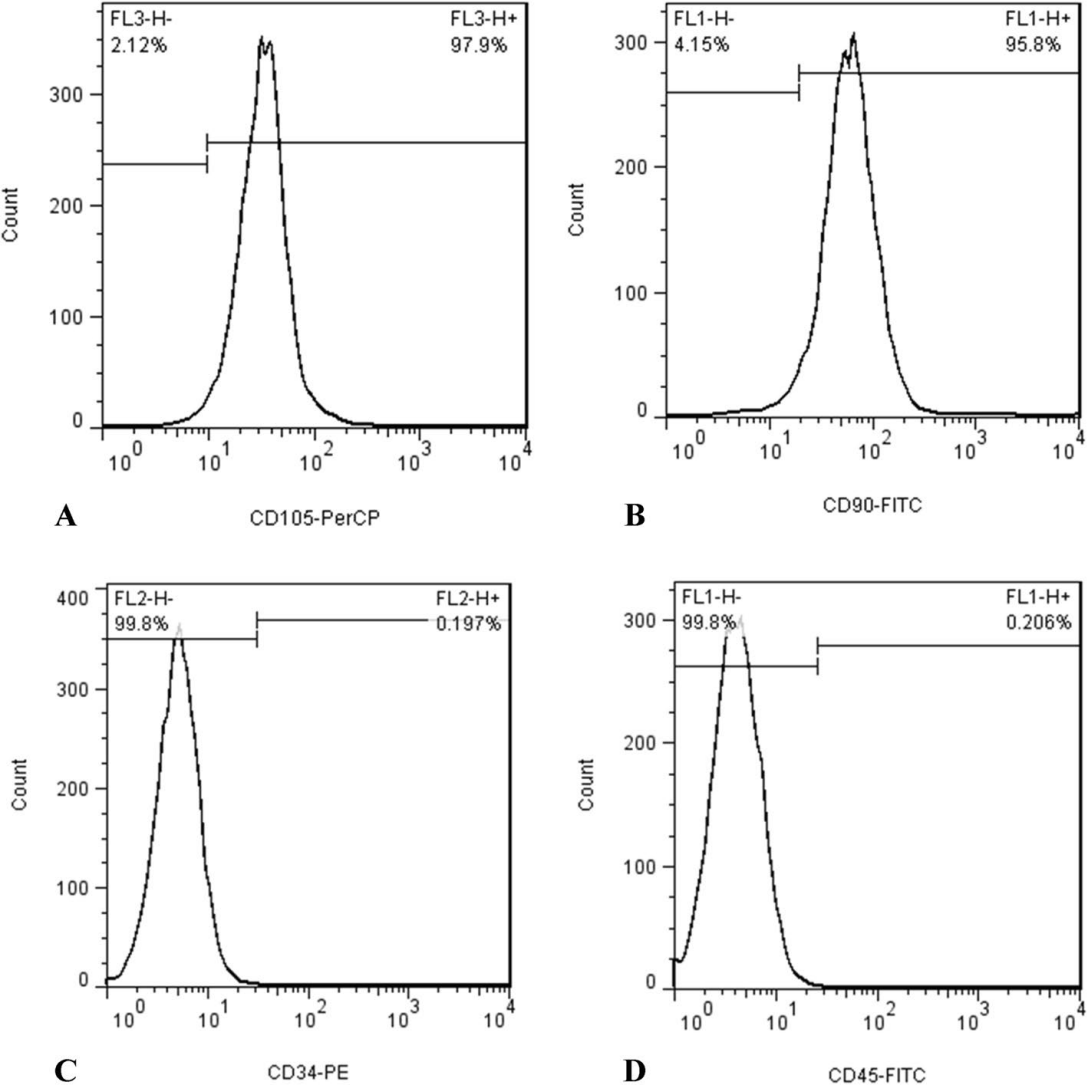
Flow cytometric results indicated a relatively high expression of the mentioned surface markers (**A**) Expression of CD105-PerCP marker, (**B**) CD90-FITC marker, (**C**) CD34-PE marker, (D) CD45-FTIC

Figure 1B presents the FTIR spectra for the synthesized CuONPs. According to the FTIR spectra of CuONPs, the most significant absorbance peak was noted at 524 cm^−1^, corresponding to Cu–O stretching vibration [38]. A strong absorbance peak at 1041 cm^−1^ was attributed to the stretching vibrations of the C-O bond in the structure of the carboxylic group and flavonoids present in the structure of the pomegranate peel extract. The peak at 1382 cm^−1^ corresponded to the stretching vibration of the C-N bond in the amine group [39]. A strong absorbance band at 1623 cm^−1^ was attributed to the bending vibration at C = C. Absorbance at 2917 cm^−1^ was attributed to asymmetric and symmetric stretching vibrations of C-H in phenolic compounds. Also, a wide band at 3402 cm^−1^ was attributed to stretching vibrations of the O–H band of the hydroxyl group [39]. The surface morphology of the synthesized CuONPs was inspected under the scanning electron microscopy (SEM). The results are presented in Fig. 1C. SEM micrographs indicated that the synthesized nanoparticles had a spherical shape with some aggregates. The isolated cells had a fibroblast-like morphology (spindle-shaped) with optimal homogeneity (Fig. 2). Flow cytometry analysis by using antibodies against CD45, CD34, CD90, and CD105 revealed stromal surface markers on the cytoplasmic membrane of the cells. Flow cytometric results indicated a relatively high expression of the abovementioned surface markers (Fig. 3).

### In vitro assessment of cell viability

**Fig. 4:**
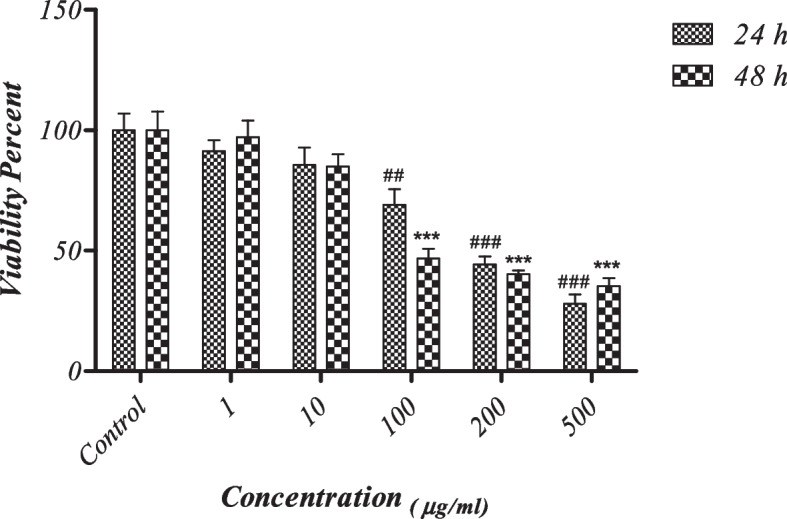
Cell viability of the groups at 24 and 48 h an increase in the concentration of CuONPs decreased the viability of SCAPs such that the viability of SCAPs exposed to different concentrations of CuONPs was significantly different at 24 and 48 h (*P* < 0.05). ***and ###: *P* < 0.001. ** and ##: *P* < 0.01. * and #: *P* < 0.05

The MTT test was used to assess the effect of CuONPs and LED irradiation on the proliferation and viability of SCAPs. As shown in Fig. 4, an increase in the concentration of CuONPs decreased the viability of SCAPs such that the viability of SCAPs exposed to different concentrations of CuONPs was significantly different at 24 and 48 h (*P* < 0.05).

The analysis revealed that the cell viability in 1 and 10 µg/mL CuONP groups was similar to the control group (*P* > 0.05). However, the viability of SCAPs exposed to 100, 200 and 500 µg/mL concentrations of CuONPs was significantly lower than that of the control group (*P* < 0.05). Thus, 1 µg/mL concentration of CuONPs was used for the next steps of the study and assessment of the expression of cell differentiation genes and proteins.

### Quantitative real-time PCR

To assess the effect of CuONPs and LED on odontogenic/osteogenic differentiation of SCAPs, the expression of osteogenic/odontogenic markers, including DSPP, BSP, ALP, and DMP1 was assessed using quantitative real-time polymerase chain reaction (PCR). The expression of each of the abovementioned genes in the experimental groups was compared with the control group.

**Fig. 5:**
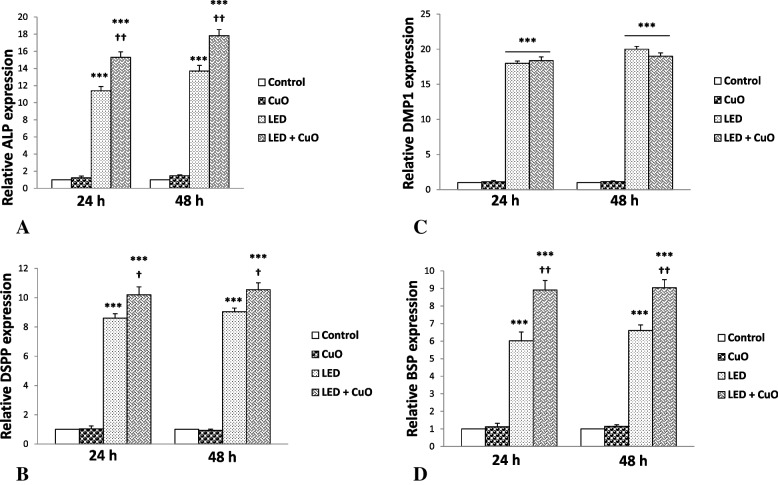
The expression profile of SCAPs treated with LED, CuONPs, and LED + CuONPs. The relative mRNA expressions of genes were compared among the three groups and also with the control group (undifferentiated SCAPs). **A**: ALP, **B**: DSPP, **C**: DMP1, **D**: BSP, after 24 and 48 h

As shown in Fig. 5, the maximum expression of BSP, ALP, and DSPP genes at 24 and 48 h was noted in the CuONPs + LED group followed by the LED group (*P* < 0.05). The minimum expression of these genes was noted in CuONPs and control groups. The expression of DMP1 after 24 and 48 h in the LED, and CuONPs + LED groups was significantly higher than that in the CuONPs and control groups (*P* < 0.05).

The maximum expression of BSP, ALP, and DSPP genes at 24 and 48 h was noted in the CuONPs + LED group, followed by the LED group (*P* < 0.05). The minimum expression of these genes was noted in CuONPs and control groups. The expression of DMP1 after 24 and 48 h in the LED, and CuONPs + LED groups was significantly higher than that in the CuONPs and control groups (*P* < 0.05).

### Alizarin red S staining

**Fig. 6:**
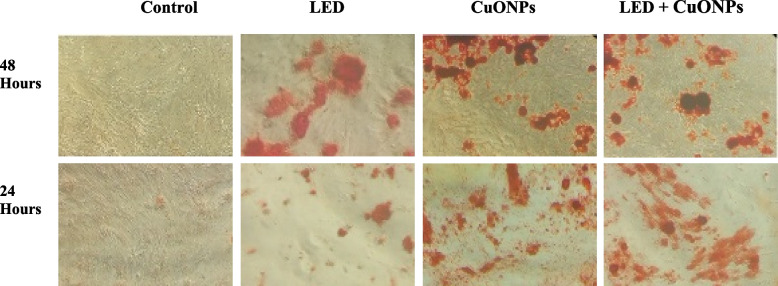
Alizarin red staining of treated SCAPs, the density of ARS increased in all experimental groups after 24 h and in CuONPs + LED and CuONPs groups after 48 h compared with the control group

Osteogenic differentiation of SCAPs treated with CuONPs and LED was evaluated qualitatively by ARS, as the final indicator of osteogenesis for assessment of matrix mineralization. As shown in Fig. 6, the density of ARS after 21 days increased in all experimental groups (24 h and 48 h of exposure to the biomaterials) compared with the control group. In other words, LED irradiation and exposure to CuONPs increased bone mineralization.

## Discussion

This study assessed the effect of CuONPs and LED irradiation on the viability and odontogenic/osteogenic differentiation of SCAPs. Considering the anatomical position of SCAPs and their important role in endodontic regenerative treatments, these cells were selected for the present study [4, 40].

Nanomaterials are defined as materials with at least one dimension in the range of 1–100 nm. The physicochemical properties of nanoparticles affect their interactions with cells and, subsequently, their overall potential toxicity [41]. Different techniques are used for the assessment of cell viability. The MTT assay is the standard technique for the assessment of cytotoxicity. According to the results of the MTT assay (Fig. 2), by an increase in the concentration of CuONPs, the viability of SCAPs decreased after 24 and 48 h, and maximum cell viability was noted with the use of 1 µg/mL CuONPs. In explanation for this finding, it should be noted that the released metal ions damage the proteins. These ions enter the structure of certain metalloproteins and deactivate them. Another mechanism of nano-toxicity is to stop the cell cycle, which is followed by cellular dysfunction or apoptosis [41]. Zhen et al. reported that CuONPs induced oxidative stress in human umbilical vein endothelial cells (HUVECs) that led to DNA damage and cell death [42]. Evidence shows that CuONPs enter human cells through endocytosis. Over time, reactive oxygen species increase in cells and decrease antioxidant enzymes. Thus, the formation of reactive oxygen species plays an important role in explaining the nano-toxicity mechanism of CuONPs [43]. In line with these findings, Zhang et al. [44] showed dose-dependent and time-dependent cytotoxicity and genotoxicity of CuONPs against mesenchymal stem cells. Also, the surface chemistry of nanoparticles affected the results to some extent [44]. Thus, 1 µg/mL concentration of CuONPs was used to assess the effect of CuONPs and LED irradiation on osteogenic/odontogenic differentiation of SCAP in the present study. Real-time PCR was used to assess the effect of CuONPs and LED irradiation on the odontogenic/osteogenic differentiation of SCAPs. As shown in Fig. 5, the highest expression of genes at 24 and 48 h was noted in the CuONPs + LED group followed by the LED group. However, the control and CuONPs groups had no significant difference in this respect. In other words, the combined use of CuONPs and LED irradiation significantly increased the expression of genes compared with the LED and control groups; however, the expression of genes following exposure to CuONPs alone had no significant difference with the control group. Several studies have assessed the effect of CuONPs and LLLT on cells. LLLT can increase the proliferation of different cell lines with no cytotoxic effects. A review of the relevant studies indicated that LLLT increases the synthesis of ATP, DNA, and RNA in stem cells and other cell lines [45]. Also, evidence shows that LLLT significantly increases bone formation and decreases inflammation with no significant effect on scaffold resorption [46]. Yi et al. reported that gold nanoparticles promoted the osteogenic differentiation of MSCs. There is evidence that gold nanoparticles might interact with proteins located in the cytoplasm, thereby interfering with certain signaling pathways within the cell. As a result of the up regulation of integrins, it is apparent that gold nanoparticles are interacting with the extracellular matrix. In both of these processes, mechanical stress is applied to the MSCs, resulting in the activation of the p38 MAPK signaling pathway, which leads to an increase in osteogenic gene expression [47]. Tang et al. showed that the formulated zinc oxide nanoparticles have possessed the notable bone regenerative activity through the enhancement of cell proliferation, differentiation, and calcium deposition in the human osteoblast-like cells [48]. In another study, silver-core shell nanowires were found to be effective in enhancing the differentiation of osteoblasts induced by reduced oxygen species by regulating the key marker genes runx2, osteoix, and osteocalcin [49].

Previous studies on the effect of LLLT on odontogenic differentiation and biomineralization of human dental pulp stem cells found that LLLT significantly increased the expression of markers related to odontogenesis [21, 50]. Also, LLLT can significantly enhance the angiogenesis and dentinogenesis of the dentin-pulp complex [51]. Considering the previous findings, the positive effect of LED irradiation on the expression of odontogenic/osteogenic markers in the present study was not far from expected. However, the synergistic effect of CuONPs combined with LED and significant upregulation of genes in the CuONPs + LED group compared with the LED group may highlight the promising effects of CuONPs in regenerative endodontics. Previous studies used copper in the composition of scaffolds; however, it was not in the form of nanoparticles. For instance, a previous study compared the viability, proliferation, attachment, and morphology of dental pulp stem cells in two scaffolds, namely Cu-doped Mg-based and Zn-doped Mg-based scaffolds. They found that dental pulp stem cells can adhere to and proliferate on both scaffolds. However, apatite formation only occurred in the Cu-doped scaffold group. Also, cell proliferation and viability decreased after 3 days, which may be due to the high concentration of copper and other released elements. Structural differences in scaffolds can explain their different bioactivity [52]. However, in the present study, the expression of genes after 48 h was still significantly higher than that in the control group, which may be due to the structure of nanoparticles used in this study. Another study discussed that incorporation of inorganic compounds into biodegradable polymer substrates is among the most applicable techniques for enhancement of proliferation, differentiation, and bio-mineralization of dental pulp stem cells, because combined synergistic effects of components result in superior mechanical and biological properties of the scaffold [53]. Similarly, in the present study, the combined use of CuONPs and LED yielded the best results.

The role of LLLT in the enhancement of photo-stimulatory and photo-biomodulatory effects, improvement of cell metabolism and regeneration, and anti-inflammatory responses has been previously confirmed [54]. As shown in Fig. 6, the density of ARS increased in all experimental groups after 24 h, and in CuONPs + LED and CuONPs groups after 48 h compared with the control group. In other words, LED irradiation and exposure to CuONPs increased bone mineralization. We observed a smaller increase in Alizarin Red staining at 48 h compared with 24 h. There may be a reason for this phenomenon, since the number of cell growth areas is limited, the cells are maturing, and the processes of bone formation by osteoblasts are shifting to the maintenance of bone by osteocytes [55, 56].

In contrast to the present findings, a previous study used ARS and Oil Red O staining to assess the osteogenic and adipogenic differentiation of mesenchymal stem cells following exposure to different types of CuONPs. They demonstrated that exposure to 10 µg/mL CuONPs had no significant effect on the differentiation potential of mesenchymal stem cells. This difference may be related to the dose-dependent and time-dependent toxicity of CuONPs [44] such that 10 µg/mL concentration had no significant effect on calcium deposition while 1 µg/mL concentration of CuONPs yielded promising results in the present study.

In spite of the fact that our study is still at the preclinical stage, in the future, our research will focus on mechanisms of action, viability of cells over a longer time, and the effect of metal nanoparticles on apoptosis and migration of stem cells. To have an accurate assessment of nanosafety, it would be necessary to develop a better understanding of the mechanisms of CuONP-associated toxicity.

## Conclusion

According to the present results, the addition of CuONPs and LED irradiation of SCAP in the culture medium significantly enhanced the osteogenic/odontogenic differentiation.

## Data Availability

The complete documentation of participant enrolled in this study belongs to the corresponding author, Hadiseh Abbaspourrokni, and is available only upon reasonable request.

## Abbreviations

SCAPs: Stem cells from the apical papilla
MTT: Methyl thiazolyl tetrazolium
ISSCR: International society for stem cells research
qrt-PCR: Quantitative reverse-transcription polymerase chain reaction
ARS: Alizarin red staining
LLL: Low level laser
LLLI: Low level laser irradiation
LLLT: Low level laser therapy
ALP: Alkaline phosphatase
DSPP: Dentin sialophosphoprotein
DMP1: Dentin matrix protein 1
BSP: Bone sialoprotein
DPSCs: Dental pulp stem cells
Hpdl: Human periodontal ligament
hPDLSCs: Human periodontal ligament stem cells
LED: Light-emitting diode
PBS: Phosphate-buffered saline
SPSS: Statistical package of the social sciences
Fig.: Figure
CuONPs: Copper oxide nanoparticles
CuO: Copper oxide
XRD: X-ray diffraction
FT-IR: Fourier-transform infrared spectroscopy
SEM: Scanning electron microscopy
